# Assessments of Wnt/JAK-STAT Signaling Pathway in Relation to Sfrp5 Among Patients with Cardiac Diseases

**DOI:** 10.3390/ijms262411943

**Published:** 2025-12-11

**Authors:** Mohammed H. Hassan, Sawsan M. A. Abuhamdah, Omyma Ashraf Hasan, Mohammed AK, Asmaa Nafady, Nehal Ashraf Zaki, Marwa Abdelhady, Rana Toghan, Tahia H. Saleem

**Affiliations:** 1 Department of Medical Biochemistry & Molecular Biology, Faculty of Medicine, Qena University, Qena 83523, Egypt; omaymaashraf2013@yahoo.com (O.A.H.); nehal.ashraf888@gmail.com (N.A.Z.); 2 Department of Biopharmaceutics and Clinical Pharmacy, School of Pharmacy, The University of Jordan, Amman 11942, Jordan; s.abuhamdah@ju.edu.jo; 3 Department of Internal Medicine, Faculty of Medicine, Qena University, Qena 83523, Egypt; mohamed_abdallah@med.svu.edu.eg; 4 Department of Clinical and Chemical Pathology, Faculty of Medicine, Qena University, Qena 83523, Egypt; asmaa_nafadyn@yahoo.com; 5 Department of Internal Medicine, Luxor University, Luxor 85951, Egypt; dr.marwa21382@gmail.com; 6 Department of Medical Physiology, Faculty of Medicine, Qena University, Qena 83523, Egypt; rana.ahmed@med.svu.edu.eg; 7 Department of Medical Biochemistry & Molecular Biology, Faculty of Medicine, Assuit University, Assuit 71515, Egypt; tahia.saleem@yahoo.com

**Keywords:** Wnt signaling, JAK-STAT pathway, SFRP5 polymorphism, cardiovascular disease, molecular biomarkers

## Abstract

Cardiovascular diseases have become a leading global health burden, with rising mortality worldwide. WNT and JAK/STAT have been highlighted as emerging biomarkers in cardiovascular disease pathogenesis. This study assessed the Wnt/JAK-STAT signaling pathway in relation to SFRP5 and genetic polymorphisms in cardiac patients. This prospective case–control study included 100 patients with various cardiac diseases (IHD, valvular heart disease, HF, cardiomyopathy, and arrhythmia) and 50 matched healthy controls. Clinical and echocardiographic assessments were performed. Plasma SFRP5, Wnt5a, and JAK levels were measured using ELISA; STAT5A expression by flow cytometry; and SFRP5 (rs780369540) gene polymorphism by TaqMan real-time PCR were also performed in all participants. Cardiac patients showed significantly higher median BMI (33 vs. 28.5 kg/m^2^, *p* = 0.001) and markedly increased median value of each Wnt5a (16.85 vs. 5.6 pg/mL, *p* < 0.001), median JAK (9.45 vs. 2.4 pg/mL, *p* < 0.001), and STAT5A expression (87.55% vs. 33%, *p* < 0.001), with lower SFRP5 levels (4 vs. 6.7 ng/L, *p* < 0.001) compared to control. The SFRP5 (rs780369540) T allele was more frequent in patients (51.5% vs. 32%, *p* = 0.001), and dominant TT + TC genotypes were higher (66% vs. 42%, *p* = 0.005) compared to the control group. TT carriers showed higher median Wnt5a, lower median SFRP5, and reduced ejection fraction compared to other genotypes (TC, CC) carriers. Multivariate analysis identified elevated Wnt5a, JAK, and decreased SFRP5 as independent predictors of cardiovascular disease (*p* < 0.05). Cardiac patients showed altered WNT5a, JAK, and SFRP5 levels. SFRP5 polymorphism predicted cardiovascular risk independently.

## 1. Introduction

Over the past decade, cardiometabolic diseases such as diabetes, stroke, and coronary heart disease (CHD) have emerged as major global health challenges due to their high prevalence and mortality. Mortality from cardiovascular diseases (CVD) has increased by nearly 60% over the last 30 years, rising from 12.1 million deaths in 1990 to 20.5 million in 2021. By 2022, CVD—particularly stroke and CHD—had become the leading cause of death and disability worldwide [1].

In parallel, the past two decades have witnessed remarkable advances in immunology that revolutionized the understanding of myocardial and vascular inflammation. These developments provided new insights into the immune processes driving the pathophysiology of cardiometabolic diseases, transforming traditional views of CVD mechanisms [2,3].

Despite this progress, the immunological aspects of cardiology were long viewed as an interdisciplinary niche. Only recently has cardiac immunology gained focused attention, bridging previously separate fields. A growing research direction now explores novel biomarkers and molecular signaling pathways—particularly WNT and JAK/STAT—that play key roles in CVD pathogenesis and enhance diagnostic and prognostic prediction [1].

SFRP5 protects the heart from ischemic injury. Clinically, low SFRP5 (or high Wnt5a/SFRP5 ratio) is associated with worse outcomes in heart failure and acute MI. SFRP5 appears to inhibit vascular calcification by interfering with osteogenic Wnt pathways [4].

JAK/STAT signaling can contribute to adverse cardiac remodeling, hypertrophy, and fibrosis. Chronic cytokine stimulation, such as through interleukin-6 (IL-6) and tumor necrosis factor-alpha (TNF-α), triggers sustained activation in both cardiac and non-cardiac tissues, leading to pathological remodeling of the myocardium. This maladaptive process includes extracellular matrix accumulation, chamber dilation, and contractile dysfunction—features commonly observed in heart failure and post-infarction syndromes [5].

However, no previous studies have investigated the interaction between SFRP5 rs4808793 and cardiometabolic markers (circulating Wnt5a-JAK/STAT) in cardiac patients, representing a significant gap in the literature. We hypothesized that the SFRP5 rs4808793 single-nucleotide polymorphism contributes to cardiac disease susceptibility through modulating Wnt and JAK/STAT molecular pathways, and that carriers of the risk genotype display distinct biochemical and molecular profiles compared to other genotypes. Also, this present study aims to investigate the possible association of blood SFRP5 levels with alterations in circulating JAK/STAT pathway markers.

## 2. Results

The median age among the cases was (61.5 years) and among the controls (54 years), with a *p*-value (0.055). Males represented (52%), and females represented (48%) among the cases, while for the control group, males represented (33%), and females (17%), with a *p*-value (0.103). Half (50%) of the cases were identified as smokers, while (31%) of the controls were smokers, with a *p*-value (0.164). The median BMI value was higher in cases (33 Kg/m^2^) than in the control group (28.5 Kg/m^2^), with a *p*-value (0.001). In total, (46%) of cases have positive family histories for cardiac diseases.

Among cardiac patients, 79% had associated comorbidities. The most common were hypertension (73%), diabetes mellitus (42%), and chronic kidney disease stages 1–3 (9%). Each cardiac subtype—ischemic heart disease, heart failure, valvular disease, arrhythmia, and cardiomyopathy—was represented equally (20% each). Regarding medications, 82% of patients were on ACE inhibitors, 80% on antihyperlipidemic drugs, and 79% on ARBs.

Among patients with valvular heart disease (n = 20), mitral regurgitation was the most prevalent lesion, identified in 6 patients (30%). Aortic stenosis was observed in 5 patients (25%), while aortic regurgitation and tricuspid regurgitation were each documented in 3 patients (15%). Mitral stenosis was present in 2 patients (10%), and pulmonary stenosis was reported in 1 patient (5%). In the cardiomyopathy cohort (n = 20), dilated cardiomyopathy constituted the majority of cases (12 patients; 60%), followed by hypertrophic cardiomyopathy in 5 patients (25%) and restrictive cardiomyopathy in 3 patients (15%). Among patients with cardiac arrhythmias (n = 20), sinus tachycardia was recorded in 10 patients (50%), atrial fibrillation in 5 patients (25%), right bundle branch block in 3 patients (15%), left bundle branch block in 1 patient (5%), and ventricular tachycardia in 1 patient (5%). Regarding heart failure cases (n = 20), heart failure with preserved ejection fraction accounted for 9 patients (45%), heart failure with reduced ejection fraction for 8 patients (40%), and heart failure with mildly reduced ejection fraction for 3 patients (15%). Within the ischemic heart disease cohort, ST-elevation myocardial infarction (STEMI) was reported in 3 patients (15%), non-ST-elevation myocardial infarction (non-STEMI) in 6 patients (30%), stable angina in 10 patients (50%), and unstable angina in 1 patient (5%).

The demographic data and comorbidities of the various groups of cardiac diseases among the included patients are presented in Table 1.

The median Fs % was lower in cases 20.6 (18.8–28.2) compared with control 30 (19.85–32), with a *p*-value (0.001). The median LVESD (cm) was higher in cases 4.35 (3.59–5.28) compared with control 3.59 (2.95–4.38), with a *p*-value (0.001). The median LVEDD (cm) was higher in cases 5.4 (5–6.68) compared with control 5.1 (4.2–5.5) with a *p*-value (0.010). The IVSD (cm) was hypertrophied in 35 cases (35%), while the median ejection fraction percentage was lower in cases 37 (32–48) compared with control 52 (37.25–55). The ejection fraction status (reduced ≤ 40) in 55%, (mild 41–49) in 24%, and (preserved ≥ 50) in 21% of cases (Table 2).

Cardiac patients showed a significantly higher median Wnt5a (16.85 vs. 5.6 pg/mL, *p* < 0.001), JAK (9.45 vs. 2.4 pg/mL, *p* < 0.001), and STAT5A expression (87.55% vs. 33%, *p* < 0.001) compared with the control group, while SFRP5 levels were significantly lower (4 vs. 6.7 ng/L, *p* < 0.001) (Table 3).

Regarding the circulating levels of the measured specific biochemical parameters in terms of the type of cardiac disease, a significantly higher median JAK concentration was observed among patients with arrhythmias, compared with those with valvular heart disease. Patients with heart failure demonstrated a significantly lower median SFRP5 concentration relative to patients with arrhythmias, with insignificant differences regarding WNT5a and STAT5A expressions between patients with different cardiac diseases (Table 4).

The genotyping of SFRP5 (rs780369540) single-nucleotide polymorphism showed significant genotype and allele differences between cardiac patients and controls. The heterozygous TC genotype was more frequent in patients (29% vs. 20%, *p* = 0.019). Dominant genotypes (TT + TC) were significantly higher in cases (66% vs. 42%, *p* = 0.005; OR = 2.681, 95% CI: 1.334–5.385), while the CC genotype was associated with lower cardiovascular disease risk.

The T allele frequency was higher in patients (51.5% vs. 32%, *p* = 0.001; OR = 2.256, 95% CI: 1.364–3.733), whereas the C allele was more common in controls, suggesting a negative association with cardiac diseases. The recessive model (TC + CC) showed no significant difference (*p* = 0.063) (Table 5).

SFRP5 levels demonstrated a negative correlation with WNT5a (r = −0.299, *p* = 0.002) and with STAT5A gate % (r = −0.200, *p* = 0.046), indicating an inverse association between SFRP5 and both WNT5a signaling activity and STAT5A expression (Table 6).

Wnt5a levels were higher in TT genotype carriers compared with TC and CC genotypes (*p* = 0.021). SFRP5 levels were lower in the TT genotype (*p* < 0.001), while STAT5A expression was higher (*p* = 0.046).

Regarding echocardiographic parameters, TT carriers showed a lower fractional shortening and ejection fraction (*p* < 0.001 for both), with higher LVESD and LVEDD values (*p* < 0.001) (Table 7).

Univariate logistic regression analysis showed that higher BMI (Exp (B) = 0.843, 95% CI = 0.760–0.936), elevated Wnt5a (Exp (B) = 0.746, 95% CI = 0.668–0.832) and JAK (Exp (B) = 0.840, 95% CI = 0.773–0.913), lower SFRP5 (Exp (B) = 2.098, 95% CI = 1.637–2.690), and reduced fractional shortening (Exp (B) = 1.110, 95% CI = 1.048–1.175) and ejection fraction (Exp (B) = 1.069, 95% CI = 1.032–1.107) were significant predictors of cardiac disease (*p* < 0.001).

In multivariate logistic regression analysis, increased BMI (Exp (B) = 0.847, 95% CI = 0.729–0.985), higher Wnt5a (Exp (B) = 0.819, 95% CI = 0.723–0.928) and JAK (Exp (B) = 0.904, 95% CI = 0.840–0.972), and decreased SFRP5 (Exp (B) = 1.443, 95% CI = 1.055–1.972) remained independent predictors (*p* < 0.05), while fractional shortening and ejection fraction lost significance.

## 3. Discussion

In our study, cardiac patients were slightly older than controls, though the difference was not statistically significant. This agrees with Ilic and Ilić [6], who found no significant age difference between myocardial infarction cases and controls (*p* = 0.499 for men, *p* = 0.869 for women). However, other studies reported significant associations between older age and cardiovascular disease. Li et al. [7] found cardiovascular patients significantly older than controls (61.2 vs. 46.5 years, *p* < 0.001), and Ilic and Ilić [6] observed an older age in heart failure patients.

A mild male predominance among cardiac patients in our study was not statistically significant, which was consistent with Taqiuddin et al. [8], who found a similar gender distribution in coronary artery disease (64% vs. 66%, *p* > 0.05). While men generally have a higher cardiovascular incidence [9], our finding suggests that gender differences may vary by region or risk profiles.

Smoking prevalence was higher among cases but without significance, similar to Ilic and Ilić [6], who found no significant difference between male cases (75.2%) and controls (68.1%, *p =* 0.238). In contrast, Hbejan [10] reported a strong association, with an odds ratio of 3.63 for myocardial infarction among male smokers, and Taqiuddin et al. [8] found significantly higher smoking rates among cardiac patients.

Our finding of a higher BMI in cardiac patients aligns with previous studies. Li et al. [7] reported higher BMI in psoriasis patients with cardiovascular disease (29.5 vs. 26.8 kg/m^2^, *p* < 0.001), while Ilic and Ilić [6] observed overweight/obesity in 76.1% of male cases versus 60.2% of controls (*p* = 0.015). Similarly, Oo et al. [11] confirmed the role of excess adiposity as a key cardiovascular risk factor.

Nearly half of our cardiac patients reported a positive family history, consistent with Chacko et al. [12], who found it in 48.8% of premature CHD cases versus 11.2% of controls (*p* < 0.001). Likewise, Ilic and Ilić [6] observed higher rates among male myocardial infarction cases (58.4%) than controls (39.8%).

Most of our patients had multiple comorbidities, predominantly hypertension, chronic kidney disease, and diabetes, consistent with the Cardiovascular–Kidney–Metabolic (CKM) syndrome [13,14]. Similar clustering was reported by Cunillera-Puértolas et al. [15], with hypertension in 83.3% and diabetes in 35.3% of CKD patients, and by Zemplényi et al. [16], who, with 70.2% hypertensive and 41.5% diabetic cases, also found CKD in about 30% of diabetics. Vijay et al. [17] demonstrated markedly higher mortality (78.6%) in patients with both diabetes and CKD versus 20.5% in heart failure alone. These findings support our results and indicate that our cohort represents a typical high-risk CKM population.

Cardiac disorders in our study—including heart failure, ischemic, valvular, arrhythmic, and cardiomyopathic diseases—were found in nearly equal proportions. While this distribution differs from global data showing ischemic heart disease and stroke as dominant causes of cardiovascular death (85%) [18], it agrees with findings from specialized referral settings, where advanced heart failure of diverse etiology predominates [19,20]. This reflects referral bias typical of tertiary heart failure clinics.

Most patients were on lipid-lowering agents and RAAS inhibitors, aligning with AHA and ESC recommendations [21,22]. Trials, such as the Heart Protection Study and SOLVD, confirmed significant mortality benefits of statins and ACE inhibitors in cardiovascular disease [23,24,25]. Hence, the high prescription rates in our cohort indicate adherence to evidence-based therapy.

ECG abnormalities, mainly atrial arrhythmia and left bundle branch block (LBBB), were frequent and typical of patients with structural heart disease. This agrees with Khan et al. [26], who found atrial fibrillation in 29% of patients with bundle branch block versus 11.8% without (*p* < 0.001), and Ashraf et al. [27], who reported LBBB in one-third of heart failure cases. Both are established predictors of adverse outcomes [28].

Cardiac patients in our study showed reduced ejection fraction and fractional shortening, with increased LVEDD, LVESD, and IVSD, indicating systolic dysfunction and ventricular dilation. Similar findings were reported by Narayanan K. et al. [29], where sudden cardiac death cases had larger LV dimensions and lower LVEF (*p* < 0.01). Such changes typify dilated cardiomyopathy and ischemic remodeling [30,31]. Conversely, HFpEF often presents with a non-dilated ventricle [32], and recent reports even suggest risk with small LV size [33,34], indicating variability across disease types.

Increased interventricular septal thickness observed in our cases agrees with reports of hypertensive heart disease [35]. Yang Y. et al. [36] also found septal hypertrophy predictive of coronary disease (HR = 1.155) and myocardial infarction (HR = 2.410).

Patients showed elevated WNT5a, JAK, and STAT5A levels with reduced SFRP5 compared to controls. The SFRP5 (rs780369540) TT and TC genotypes and T allele were more frequent among patients, while the CC genotype and C allele predominated in controls, indicating a protective effect. TT carriers exhibited higher WNT5a, STAT5A, LVESD, and LVEDD but lower SFRP5, fractional shortening, and ejection fraction, whereas CC carriers had better cardiac function, supporting the cardioprotective role of the C allele.

These findings agree with prior studies showing increased WNT5a and reduced SFRP5 in cardiovascular disease. Wang et al. [37] reported a 2.3-fold rise in myocardial WNT5a in heart failure patients (*p* < 0.01), while Kelly et al. [38] found significantly lower plasma SFRP5 in heart disease (1.25 ± 0.35 ng/mL vs. 2.46 ± 0.96 ng/mL, *p* < 0.01). However, Du et al. [39] found higher SFRP5 in acute MI, possibly reflecting a transient compensatory response. Elevated STAT5A in our patients may represent maladaptive chronic activation, though prior work identified STAT5A activation as protective in ischemic preconditioning [40,41].

Although rs780369540 has not been directly studied, our genotyping results align with animal data, which were used to support biological plausibility for SFRP5 gene involvement in cardiac remodeling and inflammatory signaling, and not to make direct translational claims due to species differences. Sfrp5-deficient mice (T allele analog) develop larger infarcts, greater apoptosis, and reduced function [42], whereas Sfrp5 overexpression (C allele analog) improves survival and LV function while reducing inflammation [4]. Kelly et al. [38] also reported that plasma SFRP5 correlated positively with LVEF (r = 0.52, *p* < 0.001), consistent with our finding that the TT genotype (low SFRP5) was linked to poorer systolic function.

Correlation analysis showed that SFRP5 correlated negatively with STAT5A, HBG, and LVESD; WNT5a correlated positively with LDL and LVESD but negatively with MPV; JAK was negatively correlated with LDL, while STAT5A correlated negatively with MPV and positively with RBC count. The inverse association between SFRP5 and LVESD agrees with Kelly et al. [38], while An et al. [43] found a positive correlation with LVEDD, possibly reflecting advanced disease variability. WNT5a’s positive correlation with LVESD supports its established role in adverse remodeling [44]. However, it is a positive association with LDL contrasts with Qin et al. [45], who showed WNT5a reduced cholesterol accumulation, suggesting context-dependent effects. The link between STAT5A and RBC count aligns with its role in erythropoiesis via the EPOR/JAK2/STAT5 pathway [46].

Univariate analysis identified high BMI, low SFRP5, elevated WNT5a and JAK, and reduced FS and EF as significant predictors of cardiac disease (*p* < 0.001). In multivariate analysis, only BMI, SFRP5, WNT5a, and JAK remained independent predictors (*p* < 0.05). This pattern parallels previous evidence that obesity and WNT/JAK dysregulation are upstream disease drivers [47,48]. Reduced FS and EF, though significant univariately, lost predictive power after adjustment, consistent with other studies [38,49], suggesting these echocardiographic indices reflect downstream manifestations of molecular and metabolic dysfunction rather than independent causal factors.

Our study identified distinct metabolic, biochemical, and genetic differences in cardiac patients, including higher BMI, elevated WNT5a and JAK, reduced SFRP5, and impaired systolic function. The SFRP5 rs780369540 polymorphism was significantly associated with disease risk, with the C allele showing lower cardiovascular disease prevalence. Multivariate analysis confirmed BMI, SFRP5, WNT5a, and JAK as independent predictors of cardiac disease, suggesting their potential as prognostic biomarkers.

The relatively small sample size for a genetic association study, especially for subgroup comparisons; the cross-sectional design, which may preclude causal inference; the single-center cohort; and the subgroup heterogeneity of the included cardiac patients were the main limitations of the current study. Additionally, the intentional stratified sampling and lack of a full-length intracellular JAK assay limit the generalizability of the findings.

## 4. Methods and Materials

This cross-sectional case–control study included 100 patients with cardiovascular disease and 50 healthy controls. The study was conducted at the Internal Medicine Department of Qena University Hospitals in collaboration with the Medical Biochemistry and Molecular Biology Department, Faculty of Medicine, Qena University, following ethical committee approval (ethical approval code SVU-MED-MBC004-2-23-8-712, approval date: 23 August 2023), over the period from 28 August 2021 to 30 August 2024. Informed, written consent was obtained from all participants prior to their recruitment in the study.

Eligible participants were adults aged 18 years or older of both sexes. The patient group included individuals diagnosed within the past year with ischemic heart disease, valvular disease, heart failure, cardiomyopathy, or arrhythmia. Exclusion criteria comprised congenital heart disease; malignancy; neurological disorders, such as stroke or epilepsy; end-stage renal disease requiring dialysis; and refusal to participate.

The sample size was determined to provide 80% statistical power to detect an odds ratio ≥ 2.0, assuming a minor allele frequency of about 0.20 at a 5% significance level using software G*Power version 3.1.9.7, including 100 cardiac patients (20 per subgroup) and 50 healthy controls, totaling 150 participants.

Participants were divided into two main groups: for the case group (n = 100), patients were divided into five groups: IHD, valvular heart disease, HF, cardiomyopathy, and arrhythmia, with 20 patients in each group; and the control group (n = 50) composed of healthy volunteers matched for age, sex, and BMI, with no history of cardiovascular disease. The distribution of the included patients resulted from an intentional, non-naturally occurring, stratified sampling method to ensure balanced representation for exploratory analyses. The controls underwent a history review to exclude prior cardiovascular events; a structured clinical evaluation, ECG screening, and renal function testing (serum creatinine and eGFR evaluation) were also performed to exclude subclinical cardiovascular or renal disease. No subjects with suggestive symptoms, ECG abnormalities, or renal impairment were included as control participants.

All subjects underwent thorough clinical evaluation, including detailed medical history (age, sex, disease onset, duration, and progression, comorbidities such as diabetes and hypertension, and medication use) and physical examination. Body mass index (BMI) was calculated using the formula weight (kg)/height (m^2^).

Diagnostic investigations included echocardiography to assess cardiac chamber dimensions, valvular function, and cardiac output. The determination of left ventricular end-diastolic diameter (LVEDD), left ventricular end-systolic diameter (LVESD), interventricular septum diameter (IVSD), fractional shortening (FS), and ejection fraction (EF) was performed using a GE Vivid S6 cardiac ultrasound (GE Medical Systems, Freiburg, Germany) and an ECG (By: CONTEC ECG MACHINE 300G, Model Name: ECG MACHINE 300G, Manufacturer: CONTEC, Country of Origin: China).

According to the 2010 American Heart Association Guidelines for Cardiopulmonary Resuscitation and Emergency Cardiovascular Care, clinical presentation, ECG assessments, and cardiac biomarkers were used to diagnose and categorize ischemic heart disease (IHD) [50].

1. Chronic Coronary Syndromes (CCS) or Stable Ischemic Heart Disease (SIHD):

* Stable Angina: Consistent chest pain or discomfort brought on by stress or physical activity usually goes away with rest or medicine (such as nitroglycerin).

2. ACS, or acute coronary syndromes:

* Unstable Angina (UA): New-onset chest pain, resting chest discomfort, or a marked deterioration of stable angina that already existed (crescendo angina). No discernible increase in cardiac markers, such as troponin, was found.

* Non-ST-Segment Elevation Myocardial Infarction (NSTEMI): ACS symptoms such as chest discomfort. ECG: May exhibit some abnormalities, such as T-wave inversion or ST-segment depression, but no persistent ST-segment elevation along with increased cardiac markers.

* The most serious type, ST-Segment Elevation Myocardial Infarction (STEMI), ECG: demonstrates increased cardiac markers and persistent ST-segment elevation.

According to the guidelines of the American Society of Echocardiography, two-dimensional (2D) and Doppler echocardiography were used to evaluate cardiomyopathy, heart failure, and valvular heart disorders [51]. According to LVEF, patients with HF were categorized as follows: LVEF was ≤40% for HFrEF (HF with decreased EF), 41–49% for HFmrEF (HF with mildly reduced EF), and ≥50% for HFpEF (HF with preserved EF) [52].

### 4.1. Biochemical and Molecular Analysis

Briefly, 7 mL of venous blood was collected from each participant. Of this, 4 mL was placed in EDTA tubes for genetic analysis and flow cytometry, and another 3 mL in EDTA tubes for plasma separation. Samples were processed under standardized conditions and stored at −80 °C.

Plasma concentrations of SFRP5, Wnt5a, and Janus kinase were measured using enzyme-linked immunosorbent assay (ELISA), using commercially available ELISA kits for SFRP5, Wnt5a, and JAK with catalog No.: BZEK2339 (intra-assay CV ≤ 8%, and inter-assay CV ≤ 10%), BZEK2330-4 (intra-assay CV ≤ 10%, and inter-assay CV ≤ 12%), and BZEK2340-48 (intra-assay CV = 5–8%, and inter-assay CV = 8–10%, respectively, supplied by Chongqing Biospes Co., Ltd., Chongqing, China. ELISA Micro Plate Reader (EMR-500, Ser No: 38363 (LABOMED, Inc.), Los Angeles, CA 90034, USA) was also used. JAK ELISA kit was used to measure soluble JAK-related immunoreactive fragments, rather than full-length intracellular JAK.

### 4.2. Flow Cytometry Analysis for STAT5A Expression

A STAT5A kit purchased from Medaysis (Enable innovation) company, Reno, NV, USA, (catalog no: MC0611-100UG, using flowcytometry Model: (BD FACSCalibur) of Becton, Dickinson and company) BD biosciences, San Jose, CA 95131, SN E97501021, made in USA) was used.

STAT5A expression was analyzed by flow cytometry (BD FACSCalibur supplied by Becton, Dickinson and Company, BD Biosciences, San Jose, CA 95131, SN E9750102, USA) according to the manufacture’s protocol, using FITC-conjugated monoclonal antibodies (catalog No. E-AB-1015, Elabcsience, Houston, TX, USA). Whole blood (100 µL) was lysed (by adding 2 mL of BD FACS^TM^ lysing solution supplied by the same flow cytometry company with catalog No. 349202, and was incubated for 10 min at room temperature in the dark), fixed (by adding 200 µL fixation buffer supplied by Elabcsience, Houston, TX, USA with catalog No. E-CK-A109A, and was incubated for 30–60 min at room temperature in the dark), and permeabilized (by adding 1 mL of 1× permeabilization buffer supplied by Elabcsience, USA with catalog No. E-CK-A109B, and was incubated for 20 min at room temperature in the dark). Phosphate buffer (pH = 7, supplied by Alpha Chemika, catalog No. AL 092000500, Mumbai, India) was used for the washing of samples after fixation and prior to permeabilization. After centrifugation at 1000× *g*, 2–5 µL of FITC-conjugated anti-STAT5A antibody was added and incubated for 20 min in the dark. Samples were resuspended in buffer and analyzed by flow cytometry. The antibody showed >95% purity, λmax at 495 nm, and εmax at 525 nm (Supplementary Figure S1).

### 4.3. DNA Extraction and Genotyping

Genomic DNA was extracted from whole blood collected in EDTA tubes using the QIAamp DNA Blood Mini Kit (QIAGEN, Venlo, The Netherlands, Hilden, Germany, Cat. No. 51104). The process involved protease digestion, lysis with Buffer AL, ethanol precipitation, and elution with 200 µL Buffer AE. DNA purity was verified using a NanoDrop spectrophotometer with acceptable purity values (A260/280 = 1.8–2.0).

Genotyping of the SFRP5 gene polymorphism (rs780369540, C/T transition) was performed using the TaqMan allelic discrimination PCR (Applied Biosystems 7500 Fast Real-Time PCR, Applied Biosystems, Carlsbad, CA, USA). Each 20 µL reaction contained 10 µL of TaqMan Genotyping Master Mix, 5 µL of DNA (10 ng/µL), 1 µL of primer/probe mix, and 4 µL of nuclease-free water. The thermal profile included initial denaturation at 95 °C for 7 min, followed by 40 cycles of 95 °C for 20 s and 59 °C for 60 s. Allelic discrimination plots were used to identify genotypes (Supplementary Figure S2).

Data were analyzed using SPSS version 27 (IBM, Chicago, IL, USA). Normality was tested by Kolmogorov–Smirnov and Shapiro–Wilk tests, revealing that all continuous variables were non-normally distributed. Continuous data were presented as median and interquartile range (IQR) for non-parametric variables and as mean ± SD for parametric ones. Group comparisons were performed using the Mann–Whitney U test for two groups and the Kruskal–Wallis H test for more than two groups, with Bonferroni correction applied for multiple comparisons. Nominal data were expressed as numbers and percentages. The Chi-square test was used when fewer than 20% of expected counts were below 5; otherwise, Fisher’s exact test was applied for 2 × 2 tables, and its extension, the Fisher-Freeman-Halton test, was used for larger tables. Spearman’s correlation test assessed relationships between non-parametric variables, and binary logistic regression analysis identified independent risk factors associated with cardiac disease. All relevant variables that showed statistical significance in the univariate analysis were entered into the multivariable regression model. Regression coefficients (B) were exponentiated to yield odds ratios (Exp(B), representing the change in odds associated with a one-unit increase in each predictor. Results are expressed as Exp (B) with 95% CIs and *p*-values. A two-tailed *p*-value < 0.05 was considered statistically significant. The studied SNP followed the Hardy–Weinberg equation.

## 5. Conclusions

Our study demonstrated that the SFRP5 rs780369540 polymorphism was strongly associated with cardiac disease risk, with the C allele appearing linked to lower disease susceptibility and the T allele linked to adverse cardiac markers. BMI, SFRP5, WNT5a, and JAK have been confirmed to be independent predictors of cardiac disease, highlighting their potential as biomarkers for risk stratification. The findings of the present research support the lack of the beneficial blocking effect of SFRP5 on the WNT signaling pathway, as evidenced by its low concentration, which exerts a protective effect on vascular endothelium and reduces chronic inflammation. Larger studies are needed to validate the SFRP5 single-nucleotide polymorphism (rs780369540) in broader populations and to discuss the mechanistic association between SFRP5 levels/SNP (rs780369540), JAK/STAT, and cardiac disease prognosis.

## Figures and Tables

**Table 1:** Demographic characteristics and co-morbidities of cardiac diseases’ subgroups.

Variables	Heart Failure	Ischemic Heart Disease	Valvular Heart Disease	Arrhythmia	Cardiomyopathy
	n = 20	n = 20	n = 20	n = 20	n = 20
**Age (years)**					
Min.–Max.	23–77	47–77	35–63	38–75	33–80
Median (Q1–Q3)	61 (55.25–62.75)	70 (66–75)	48.5 (36–60)	65 (55–70)	51.5 (40–66)
**Gender (NO, %)**					
Male	12 (60%)	16 (80%)	8 (40%)	8 (40%)	8 (40%)
Female	8 (40%)	4 (20%)	12 (60%)	12 (60%)	12 (60%)
**BMI (Kg/m^2^)**					
Min.–Max.	27–37	26–38	26–37	27–36	25–42
Median (Q1–Q3)	30 (27.25–32.75)	34 (33–37)	31 (29–34)	33.5 (29–36)	31 (27–33)
**Smoking (NO,%)**					
Negative	10 (50%)	4 (20%)	12 (60%)	12 (60%)	12 (60%)
Positive	10 (50%)	16 (80%)	8 (40%)	8 (40%)	8 (40%)
**Family history (NO,%)**					
Negative	20 (100%)	12 (60%)	6 (30%)	8 (40%)	8 (40%)
Positive	0 (0%)	8 (40%)	14 (70%)	12 (60%)	12 (60%)
**Associated co-morbidities (NO. %)**					
No	4 (20%)	3 (15%)	4 (20%)	5 (25%)	5 (25%)
Yes	16 (80%)	17 (85%)	16 (80%)	15 (75%)	15 (75%)
**Types of co-morbidities**					
**Hypertension (NO. %)**					
No	5 (25%)	5 (25%)	4 (20%)	5 (25%)	8 (40%)
Yes	15 (75%)	15 (75%)	16 (80%)	15 (75%)	12 (60%)
**CKD (NO. %)**					
No	18 (90%)	18 (90%)	20 (100%)	18 (90%)	17 (85%)
Yes	2 (10%)	2 (10%)	0 (0%)	2 (10%)	3 (15%)
**COPD (NO. %)**					
No	17 (85%)	18 (90%)	20 (100%)	20 (100%)	20 (100%)
Yes	3 (15%)	2 (10%)	0 (0%)	0 (0%)	0 (0%)
**DM (NO. %)**					
No	14 (70%)	6 (30%)	12 (60%)	12 (60%)	14 (70%)
Yes	6 (30%)	14 (70%)	8 (40%)	8 (40%)	6 (30%)
**Medications (NO. %)**					
**Anti-hyperlipidemic**					
No	6 (30%)	1 (5%)	0 (0%)	6 (30%)	7 (35%)
Yes	14 (70%)	19 (95%)	20 (100%)	14 (70%)	13 (65%)
**ARBs (angiotensin-receptor blockers)**					
No	7 (35%)	1 (5%)	0 (0%)	6 (30%)	7 (35%)
Yes	13 (65%)	19 (95%)	20 (100%)	14 (70%)	13 (65%)
**ACE inhibitors (Angiotensin-converting enzyme)**					
No	4 (20%)	1 (5%)	0 (0%)	6 (30%)	7 (35%)
Yes	16 (80%)	19 (95%)	20 (100%)	14 (70%)	13 (65%)

**Table 2:** Echo parameters of the studied groups.

Variables	Cases (n = 100)	Control (n = 50)	*p*-Value
**FS %**			
Min.–Max.	11–35.3	16.47–34	
Median (Q1–Q3)	20.6 (18.8–28.2)	30 (19.85–32)	<0.001 ** U
**LVESD (cm)**			
Min.–Max.	2.3–6.3	2.3–5.85	
Median (Q1–Q3)	4.35 (3.59–5.28)	3.59 (2.95–4.38)	<0.001 ** U
**LVEDD (cm)**			
Min.–Max.	3.6–7.6	3.6–7	
Median (Q1–Q3)	5.4 (5–6.68)	5.1 (4.2–5.5)	0.010 * U
**IVSD (interventricular septum diameter) (cm)**			
Normal	65 (65%)	50 (100%)	
Hypertrophy	35 (35%)	0	-
**ECHO: Ejection fraction %**			
Min.–Max.	20–60	28–59	
Median (Q1–Q3)	37 (32–48)	52 (37.25–55)	<0.001 ** U
**ECHO: Ejection fraction status**			
Reduced ≤40	55 (55%)		
Mild 41–49	24 (24%)		
Preserved ≥50	21 (21%)		

The data were presented as median and Interquartile range (Median (IQ)) for non-parametric data, Abbreviations: *p*-value—comparison between the two studied groups using the Mann–Whitney U test “U” for nonparametric continuous data. *p*-value > 0.05 considered statistically not significant, * *p*-value < 0.05 considered statistically significant, ** *p* < 0.01 considered highly statistically significant.

**Table 3:** Comparison of the measured specific biochemical parameters among the studied groups.

Variables	Cases (n = 100)	Control (n = 50)	*p*-Value
**WNT5a (pg/mL)**			
Min.–Max.	4.8–32	1.9–21	
Median (Q1–Q3)	16.85 (6.9–22)	5.6 (3.5–8.4)	<0.001 ** U
**JAK (pg/mL)**			
Min.–Max.	0.7–41.2	0.7–71	
Median (Q1–Q3)	9.45 (5.6–16.5)	2.4 (1.4–3.4)	<0.001 ** U
**SFRP5 (ng/L)**			
Min.–Max.	0.7–9.2	3.4–8.9	
Median (Q1–Q3)	4 (2.75–5.28)	6.7 (5.4–7.83)	<0.001 ** U
**STAT5A gate %**			
Min.–Max.	51.5–99	24–37	
Median (Q1–Q3)	87.55 (79.9–92.6)	33 (29.3–35.7)	<0.001 ** U

The data were presented as median and interquartile range (Median (IQ)) for non-parametric data. Abbreviations: *p*-value—comparison between the two studied groups using the Mann–Whitney U test “U” for nonparametric continuous data, *p*-value > 0.05 considered statistically not significant, ** *p* < 0.01 considered highly statistically significant.

**Table 4:** Profile of the measured specific biochemical parameters between cardiac disease subgroups.

Variables	Patients’ Subgroups					
	Heart Failure	Ischemic Heart Disease	Valvular Heart Disease	Arrhythmia	Cardiomyopathy	*p*-Value
	n = 20	n = 20	n = 20	n = 20	n = 20	
**WNT5a (pg/mL)**						
Min.–Max.	5.2–29	6–28.7	9.5–32	6.8–27	4.8–25.9	
Median (Q1–Q3)	6.7 (5.45–21)	20.2 (15–22)	16.1 (14.5–22.1)	17.45 (12.7–23)	16.15 (6.9–17.2)	0.686 ^H^
**JAK (pg/mL)**						
Min.–Max.	4.2–23	4.2–23.7	3.5–30	3.6–41.2	0.7–37	
Median (Q1–Q3)	9.35 (5.6–14.3)	7.55 (6.3–15.7)	6.8 (4.9–9.2)	16.95 (9.7–22.1)	10.85 (3.5–18.3)	0.049 * ^H^
**SFRP5 (ng/L)**						
Min.–Max.	2.1–8.4	2.4–8.9	1.14–9.2	2.2–8.1	0.7–6.7	
Median (Q1–Q3)	3.4 (2.35–4.43)	3.95 (2.78–5.25)	3.65 (2.5–5.7)	4.8 (3.45–7.1)	4 (3.6–4.9)	0.047 * ^H^
**STAT5A gate %**						
Min.–Max.	65.6–94.3	68.4–97.2	52.6–96.5	51.5–99	73.1–96.1	
Median (Q1–Q3)	89.4 (83.2–91.93)	87.55 (83.4–94.5)	84 (76.4–92.6)	86.95 (85.4–91.3)	87.6 (78.1–94.4)	0.787 ^H^

The data were presented as median and interquartile range (Median (IQ)) for non-parametric data. Abbreviations: *p*-value—comparison between all the studied groups using the Kruskal–Wallis 1-Way ANOVA (K samples) test “H” for nonparametric continuous data. *p* value > 0.05 considered statistically not significant, * *p*-value < 0.05 considered statistically significant.

**Table 5:** Frequencies of genotypes and alleles of SFRP5 SNP (rs780369540) among the studied groups.

Variables	SFRP5 GENE (rs 780369540) (No., %)							SFRP5 GENE Alleles (No., %)	
	TT	TC	CC	(TT + TC)	CC	TT	(TC + CC)	T	C
**Cases (n = 100)**	37 (37%)	29 (29%)	34 (34%)	66 (66%)	34 (34%)	37 (37%)	63 (63%)	103 (51.5%)	97 (48.5%)
**Control (n = 50)**	11 (22%)	10 (20%)	29 (58%)	21 (42%)	29 (58%)	11 (22%)	39 (78%)	32 (32%)	68 (68%)
***p*-value**	0.019 * (7.954)			0.005 ** (7.8182)		0.063 (3.447)		0.001 ** (10.242)	
**OR (95% CI)**	-			2.681 (1.334–5.385)		2.082 (0.952–4.554)		2.256 (1.364–3.733)	

The data were presented as a number (percentage). Abbreviations: *p*-value—comparison between the two studied groups using the Chi-square test; OR—odds ratio; CI—confidence interval. *p*-value > 0.05 considered statistically not significant, * *p*-value < 0.05 considered statistically significant, ** *p* < 0.01 considered highly statistically significant.

**Table 6:** Specific biochemical parameters correlation coefficients matrix among cases (n = 100).

		SFRP5 (ng/L)	WNT5a (pg/mL)	JAK (pg/mL)	STAT5A Gate
SFRP5 (ng/L)	R	1.000			
	P				
WNT5a (pg/mL)	R	−0.299 **	1.000		
	P	**0.002**			
JAK (pg/mL)	R	0.007	−0.062	1.000	
	P	0.943	0.537		
STAT5A gate %	R	−0.200 *	0.161	0.076	1.000
	P	**0.046**	0.109	0.451	

*p*-value > 0.05 considered statistically not significant, * *p*-value < 0.05 considered statistically significant, ** *p* < 0.01 considered highly statistically significant.

**Table 7:** Serum level of SFRP5 among cardiac patients according to different genotypes (rs 780369540).

Variables	SFRP GENE % (No. and %)			*p*-Value
	TT	TC	CC	
	n = 37	n = 29	n = 34	
**WNT5a (pg/mL)**				
Min.–Max.	5.2–25.9	5.3–29	4.8–32	
Median (Q1–Q3)	20 (6.85–22)	11.5 (6.5–17)	17.2 (11.88–24.1)	0.021 * H
**JAK (pg/mL)**				
Min.–Max.	3.18–41.2	3.5–23	0.7–37	
Median (Q1–Q3)	13.9 (5.6–18.9)	10.4 (6.3–12.25)	8.7 (5.28–15.7)	0.521 H
**SFRP 5 (ng/L)**				
Min.–Max.	0.7–8.9	3.3–7.5	1.5–9.2	
Median (Q1–Q3)	2.6 (2.2–4)	4.8 (3.7–5.35)	4.6 (3.58–5.7)	<0.001 ** H
**STAT5A gate%**				
Min.–Max.	68.4–99	65.6–96.5	51.5–96.1	
Median (Q1–Q3)	89.7 (84.75–92.6)	88.4 (80.3–94.3)	84.3 (73.2–92.03)	0.046 * H
**Echo parameters**				
**FS %**				
Min.–Max.	14.7–28.2	16.47–35.3	11–34.7	
Median (Q1–Q3)	18.8 (17.6–22.35)	20.6 (18.2–26.5)	31.85 (20–33.13)	<0.001 ** H
**LVESD (cm)**				
Min.–Max.	2.8–6.14	2.3–5.85	2.3–6.3	
Median (Q1–Q3)	4.56 (4.18–5.6)	4.2 (3.75–5.45)	3.55 (2.58–4.56)	<0.001 ** H
**LVEDD (cm)**				
Min.–Max.	3.9–7.2	4.6–7	3.6–7.6	
Median (Q1–Q3)	5.6 (5.3–6.8)	5.4 (5.1–6.75)	5.05 (4–5.53)	<0.001 ** H
**Ejection fraction %**				
Min.–Max.	25–48	28–60	20–59	
Median (Q1–Q3)	33 (30–41.5)	36 (31.5–46)	54.5 (37–56.25)	<0.001 ** H

*p*-value—comparison between all SFRP5 genotypes using Kruskal–Wallis 1-Way ANOVA (K samples) test “H”. *p*-value > 0.05 considered statistically not significant, * *p*-value < 0.05 considered statistically significant, ** *p* < 0.01 considered highly statistically significant.

## Data Availability

The datasets used and/or analyzed during the current study are available from the corresponding author upon reasonable request, after obtaining the permission of our institute.

## Supplementary Materials

The following supporting information can be downloaded at: https://www.mdpi.com/article/10.3390/ijms262411943/s1.
