# Supplementary material for: Assessments of Wnt/JAK-STAT Signaling Pathway in Relation to Sfrp5 Among Patients with Cardiac Diseases
Source: Int J Mol Sci. 2025 Dec 11;26(24):11943. doi: 10.3390/ijms262411943 (PMC12732818; doi:10.3390/ijms262411943)
Supplement: Supplementary file 1 [file ijms-26-11943-s001.zip › ijms-4005542-supplementary.pdf]

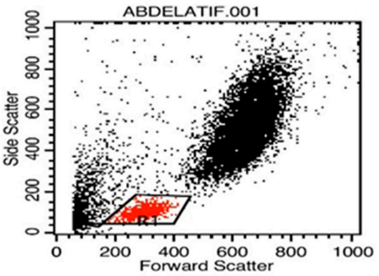

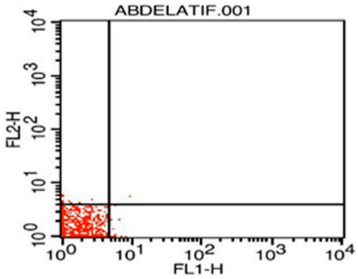

File: ABDELATIF.001

Quad	Events	% Gated	% Total
UL	13	1.69	0.13
UR	1	0.13	0.01
LL	741	96.36	7.41
LR	14	1.82	0.14

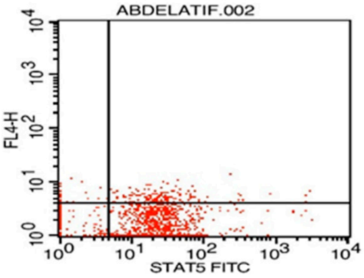

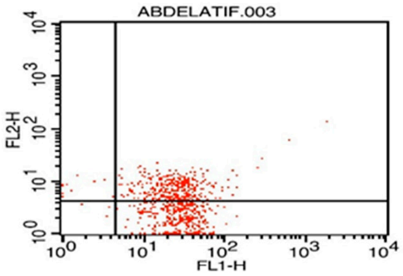

File: ABDELATIF.002

Quad	Events	% Gated	% Total
UL	22	2.06	0.22
UR	160	14.97	1.60
LL	109	10.20	1.09
LR	778	72.78	7.78

File: ABDELATIF.003

Quad	Events	% Gated	% Total
UL	10	1.36	0.10
UR	357	48.44	3.57
LL	4	0.54	0.04
LR	366	49.66	3.66

Supplementary Fig.1.Flowcytometric analysis of STAT5A expressions

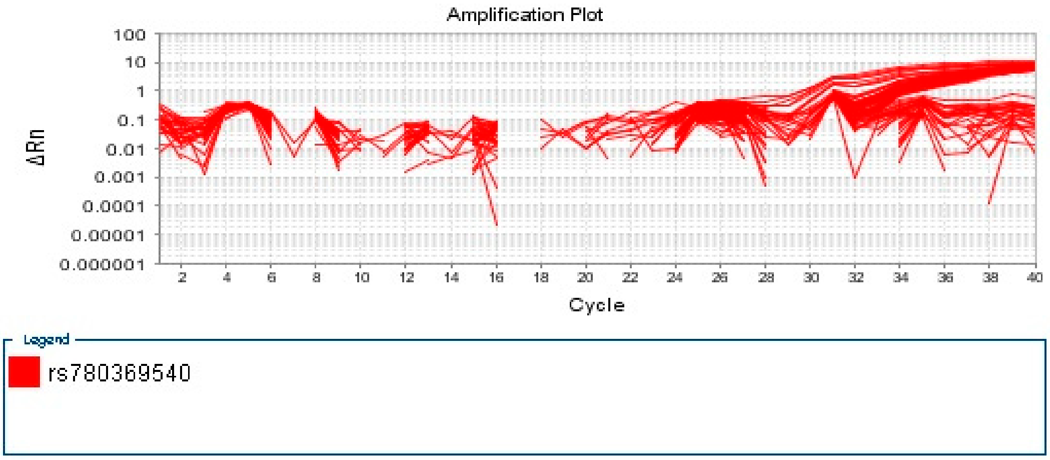

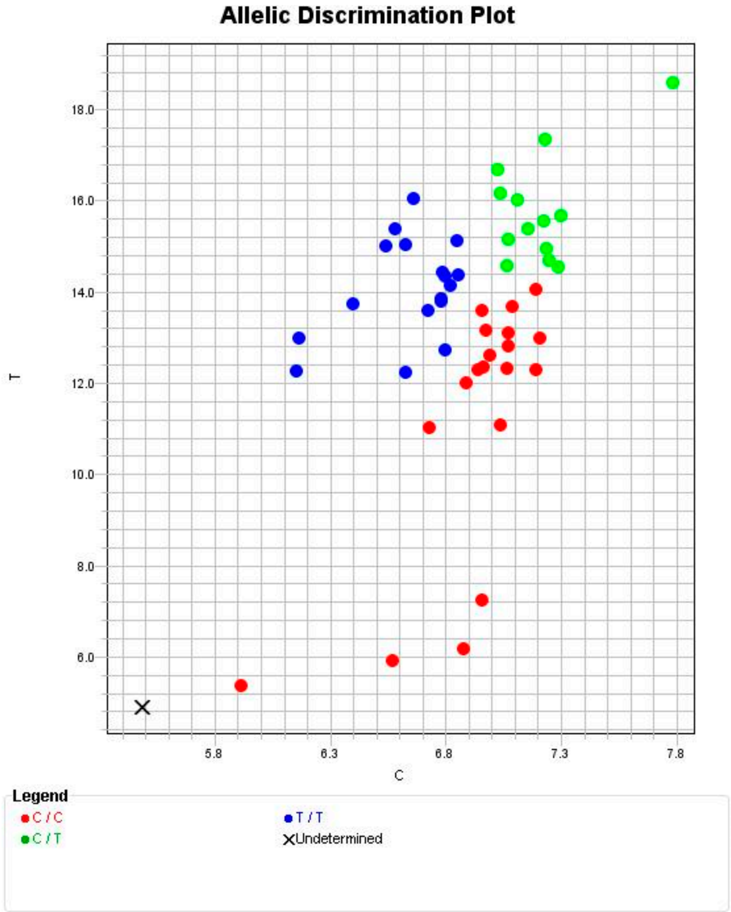

Supplementary Fig.2.Amplification and alleleic discrimination plots of SFRP5 gene polymorphism (rs780369540, C/T transition) using real time PCR technique.
